# Blockade of mTOR ameliorates IgA nephropathy by correcting CD89 and CD71 dysfunctions in humanized mice

**DOI:** 10.1371/journal.pone.0318581

**Published:** 2025-10-07

**Authors:** Alexandra Cambier, Jennifer Da Silva, Julie Bex, Fanny Canesi, Lison Lachize Neanne, Aurélie Sannier, Hélène Mathieu, Erwan Boedec, Amandine Badie, Renato C. Monteiro

**Affiliations:** 1 Service de Néphrologie pédiatrique-hémodialyse, CHU Sainte Justine, Montréal, Canada; 2 Axe Maladies immunitaires et cancer, Centre de Recherche Azrieli, Université de Montréal, CHU Sainte Justine, Montréal, Canada; 3 Centre d’expertise sur les maladies rares médiées par le complément, CHU Sainte Justine, Montréal, Canada; 4 Université de Paris Cité, Centre de Recherche sur l’Inflammation (CRI), INSERM U1149 & CNRS E8252, Inflamex Laboratory of Excellence, Paris, France; 5 Université de Paris Cité, Service anatomopathologie, Bichat Hospital, Assistance Publique Hôpitaux de Paris, Paris, France; 6 Université de Paris Cité, Institute of Psychiatry and Neuroscience of Paris (IPNP), INSERM UMR1266, Biochemistry and Biophysics facility, Paris, France; Peking University First Hospital, CHINA

## Abstract

**Background and hypothesis:**

IgA nephropathy (IgAN) is the most common glomerulonephritis and major cause of renal failure worldwide. Its pathogenesis involves galactose deficient IgA1-immune complexes containing soluble IgA Fc receptor (sCD89). Both IgA1 and sCD89 bind independently to the transferrin receptor (TfR1, CD71), a mesangial IgA1 receptor. Here, we hypothesize that sCD89 plays a pathogenic role in IgAN by driving a tri-partite IgA1-sCD89-CD71 complex inducing activation *in situ* of the mTOR (mammalian target of rapamycin) signaling pathway in mesangial cells and contributing to disease progression. mTOR inhibition may disrupt this pathogenic axis, reducing IgA1 and sCD89 deposits, modulating CD71 expression, and alleviating disease manifestations. Here, we investigated the role of sCD89 and a mTOR inhibitor using humanized mouse models of IgAN expressing CD89 and/or IgA1.

**Methods:**

Single cell and RNAseq data were obtained from public IgAN dataset and immunostaining was performed on childhood IgAN (cIgAN) biopsies. Human mesangial cells (HMCs) stimulation by recombinant sCD89 (rsCD89) was followed by western blot analysis. Pre-clinical assays with mTOR inhibitor (Everolimus) by oral gavage were performed using young α1^KI^ mice injected with rsCD89 for 25 days (preventive protocol) and adult α1^KI^CD89^Tg^ mice (treated protocol) for 75 days. Proteinuria, renal function, and circulating immune complexes (CICs) were analyzed and kidneys were harvested for histology.

**Results:**

RNAseq revealed increased TfR1 and mTOR expression in mesangial cells from IgAN patients. TfR1 upregulation was confirmed in cIgAN biopsies. sCD89 stimulation induced HMC TfR1 expression and phosphorylation of mTOR, Akt and S6K1. Everolimus treatment prevented or reverted mesangial IgA1 and C3 deposits and also decreased mesangial TfR1 and cell proliferation. Everolimus impaired levels of sCD89- and IgA-CIC, proteinuria, as well as renal function.

**Conclusion:**

These findings highlight the critical role of the sCD89-TfR1-mTOR axis in IgAN pathogenesis and support the use of mTOR inhibitors as a novel therapeutic approach. This approach could significantly improve outcomes by slowing disease progression and minimizing the systemic toxic effects of current immunosuppressive therapies. This is particularly crucial for pediatric patients, where the only approved treatment – steroids – has severe side effects, including detrimental impacts on bone health and growth.

## Introduction

IgA nephropathy (IgAN) is an autoimmune glomerular disease associated with environmental factors and genetic polymorphisms leading to a significant health burden [1,2]. It usually progresses over 20 years to end-stage renal disease (ESRD) in one third of patients [3–5], requiring kidney transplantation early in life [6–9].

IgAN pathogenesis presents four consecutive hits: (1) generation of abnormal galactose-deficient IgA1 (Gd-IgA1) [10,11], (2) an auto-immune reaction generating IgG autoantibodies targeting Gd-IgA1, (3) formation of circulating IgA1 immune complexes (CICs) composed of IgG anti-Gd-IgA1 and other components [12]. Gd-IgA1 triggers the myeloid IgA Fc receptor CD89 leading to its shedding from the cell surface [13–15]. Soluble forms of CD89 (sCD89) generate and amplify the size of CICs [14] forming larger Gd-IgA1-sCD89 CICs [16–21]. (4) The Gd-IgA1-sCD89 CIC specifically deposits in the glomerular mesangium and, at least partially through interactions with the transferrin receptor 1 (TfR1 also known as CD71), a mesangial IgA1 receptor, activates the complement cascade and glomerular inflammatory response [22]. CIC activation of human mesangial cells (HMCs) enhances cell proliferation with mesangial matrix expansion and pro-inflammatory cytokine secretion. In turn, HMC activation induces podocyte hypertrophy or sclerosis leading to proteinuria and hematuria and thus the progression of IgAN [23–25].

Mesangium is one of the main sites for immune-complex deposition resulting in cell proliferation and increased extracellular matrix production during glomerulonephritis development. In IgAN, mechanisms of proliferation of HMCs are poorly understood. TfR1 has been described as an IgA1 receptor in IgAN but also in celiac disease [26,27]. TfR1 is overexpressed in the mesangium of IgAN patients, and it is associated with IgA deposits [28]. Deglycosylation of IgA1 increases the TfR1-IgA interaction [29] and HMC stimulation by polymeric IgA1 enhances TfR1 expression on these cells, leading to a positive feedback loop that favors IgA1 glomerular deposition [30].

The mechanism by which IgA1 induces TfR1 overexpression in mesangial cells remains poorly understood. It has been demonstrated that mesangial TfR1 overexpression in IgAN is influenced not only by Gd-IgA1 but also by sCD89 and transglutaminase 2. Among these, sCD89 plays a pivotal role in the selective deposition of IgA1 in the mesangium, as evidenced by studies in a humanized mouse model expressing IgA1 either alone or in conjunction with CD89 [21,31]. In a recent study, we identified various forms of sCD89 in the serum of patients with childhood IgAN (cIgAN), with or without association with IgA1 deposits in the mesangium [21]. Patient-derived and recombinant sCD89 forms significantly activate HMCs and induce their proliferative state through interaction with TfR1 [21]. This interaction activates the PI3K/Akt/mTOR signaling pathway, which is associated with a poor prognosis in IgAN [21,31,32].

The PI3K/Akt/mTOR signaling pathway accelerates the cell life cycle, reduces apoptosis, and promotes cell migration [33,34]. mTOR inhibitors can represent an attractive therapeutic option since oral corticosteroids are the only approved immunosuppressor but have considerable systemic toxic effects, especially in pediatrics [35]. In this study, we hypothesize that sCD89 directly interacts with TfR1 to activate the PI3K/Akt/mTOR signaling pathway, driving mesangial cell proliferation and contributing to IgAN progression. Inhibiting mTOR could disrupt this pathogenic axis, reducing mesangial hyperplasia, immune complex deposition, and disease severity, thereby offering a promising therapeutic strategy for IgAN.

## Materials and methods

### Mouse procedures

Mice expressing the IgA1 heavy chain (α1^KI^) and transgenic for the human Fc α receptor I (CD89^Tg^), which spontaneously develop IgAN at 12 weeks of age as previously described (α1^KI^CD89^Tg^ mice [31]), were used as indicated. We also utilized α1^KI^ mice expressing human IgA1 [36], which were injected with recombinant sCD89 (rsCD89) over a 4-week period. Mice were weaned at 4 weeks of age, housed under specific pathogen–free (SPF) conditions, provided sterile water to drink (supplemented as indicated), and fed sterilized chow ad libitum. Ethical approval for animal experiments was granted by the local animal ethics committee (Autorisation de Projet Utilisant des Animaux à des Fins Scientifiques, APAFIS no. 42753). Mice were anaesthetized before terminal collection of blood from the retro-orbital sinus and then euthanized by cervical dislocation. Euthanized mice were perfused with PBS. Segments of kidney were snap-frozen in optimal cutting temperature (OCT) medium using liquid nitrogen. Kidneys were also fixed in 4% formaldehyde. Segments of kidney were snap-frozen dry in cryotubes using liquid nitrogen.

### Experimental procedures

For each protocol, 6 α1^KI^ mice (that received rsCD89 injection) and 20 α1^KI^CD89^Tg^ mice were used to make two arms: a treated arm receiving the mTOR inhibitor everolimus at standard dose (2 mg/kg) and a control arm receiving placebo treatment with water, administrated by daily intragastric gavage for each mouse model. The dosage was reassessed with the mice’s weight. Everolimus was dissolved in sterile water before administration by tube feeding. A first blood sample was taken before any intervention to measure the kidney function of the mice as well as the level of CICs. Urine was collected to monitor proteinuria before and after treatment. After 25 days for the α1^KI^ (with sCD89 injection) and 75 days for α1^KI^CD89^Tg^, the mice were sacrificed. Plasma and serum were collected to measure renal function and CICs. The CICs (IgA-IgG, sCD89-IgA), Gd-IgA1 and sCD89, were assessed by sandwich ELISA as described before [21]. The kidneys were harvested for histological analysis, including: (1) Ki67 staining to evaluate kidney hypercellularity, (2) assessment of human IgA1 and complement C3 deposits by immunofluorescence, and (3) evaluation of transferrin receptor expression (anti-CD71 antibody) and CD89 expression (A3 monoclonal anti-CD89 antibody) by immunohistochemistry. Quantification of immunostainings was performed in 10 glomeruli per mouse at the end of the treatment. Each dot in the plots represents a single glomerular measurement.

### Production of soluble proteins

The recombinant soluble CD89 (rsCD89) was produced at the Biochemistry and Biophysics (B&B) facility (Paris, France). Briefly, the human sCD89 sequence was codon-optimized (GeneArt, ThermoFisher, USA) to include an N-terminal His tag and TEV cleavage site and then inserted into the pCDNA3.4 vector (ThermoFisher, USA) using the NEBuilder HiFi DNA Assembly Kit (New England Biolabs, USA). The resulting pCDNA3.4-sCD89 plasmid was purified using the NucleoBond Xtra Maxi EF Kit (Macherey-Nagel, Germany) and verified by sequencing (Eurofins Genomics, Germany). The pCDNA3.4-sCD89 plasmid was transfected into Expi293 cells (ThermoFisher, USA) at a density of 3 × 10⁶ cells/mL using ExpiFectamine (ThermoFisher, USA). The cells were incubated for 6 days at 37°C, 5% CO₂, and 125 rpm. After incubation, the supernatant containing rsCD89 was centrifuged at 6,000 × g for 15 minutes and purified using a HisTrap Excel column (Cytiva, Sweden) on an ÄKTA Pure chromatography system (Cytiva, Sweden). Elution was performed with an imidazole gradient, and the rsCD89 was desalted into PBS 1X using a HiPrep 26/10 desalting column (Cytiva, Sweden).

### Cell culture and treatments

Human mesangial cells (HMCs, Innoprot #P10661) were obtained from healthy human kidney tissue. Cells were cultured at 37°C in a humidified environment containing 5% CO_2_, in Roswell Park Memorial Institute (RPMI) medium supplemented with 20% of Fetal Bovine Serum (FBS), 50 U/mL penicillin and 50 µg/mL streptomycin. Cells were used for experiments between passages 4–10. HMCs were serum deprived for 24 hours for RT-qPCR and 48 hours (medium replaced at 48, 42, 24, and 2 hours) for western blot before stimulation using RPMI supplemented with 0.5% FBS and antibiotics. Stimulations were done in RPMI 0% FBS and antibiotics, using cells cultured in 6-well plate for various durations depending on mRNA and protein assays. Albumin (25 µg/mL), β-synuclein (15 µg/mL) [21](control recombinant protein) and HumanKine® recombinant platelet derived growth factor bb (PDGF-bb #HZ-1308, 50 ng/mL) were used as negative and positive controls, respectively. Recombinant human soluble CD89 (rsCD89) was produced by the biochemistry and biophysics facility, Inserm U1266, Paris, France, and used at different concentrations. Everolimus (10µM) was used to block mTOR pathway in HMCs one hour before stimulation.

### Western blot analysis

Cells were lysed in RIPA buffer (89900, Thermo Scientific™) supplemented with protease inhibitors (cOmplete™, Roche) and phosphatase inhibitors (P0044, Sigma-Aldrich). Proteins were denatured in Laemmli buffer (BIO-RAD) containing 2.5% 2-Mercaptoethanol and 20–30 μg of proteins was loaded onto 4%−12% SDS-PAGE gels. Proteins were transferred to Low Fluorescence PVDF membranes (BIO-RAD) and detected using the following primary antibodies: mouse monoclonal anti human-TfR1 (ab269513, Abcam, 1:5,000), mouse monoclonal anti-phospho Akt (Ser473, 66444–1-Ign, Proteintech, 1:5,000), rabbit polyclonal anti-Akt (9272, Cell Signaling, 1:1,000), mouse monoclonal anti-phospho p70S6K1 (Thr389, 9206, Cell Signaling, 1:1,000, also recognizing phosphorylated p85S6K1 at Thr412), rabbit monoclonal anti-p70S6K1 (2708, Cell Signaling, 1:1,000), rabbit monoclonal anti-phospho mTOR (Ser2448, 5536, Cell Signaling, 1:1,000), and mouse monoclonal anti-mTOR (4517, Cell Signaling, 1:1,000). GAPDH was used as a protein loading control with a mouse monoclonal anti-GAPDH antibody (AM4300, Invitrogen, 1:15,000). HRP-conjugated secondary antibodies (Invitrogen) included goat anti-mouse IgG HRP (31430, Invitrogen) and goat anti-rabbit IgG HRP (31460, Invitrogen). Signals were detected using ECL kits (Thermo Scientific™, BIO-RAD) and visualized using the ChemiDoc MP System (BIO-RAD).

### Immunohistochemistry of TfR1 in cIgAN and control patients

Immunostaining was performed on formalin-fixed, paraffin-embedded section using a mouse anti-human TfR1 (ab269513, Abcam, 1:4,000), followed by detection with a polyclonal anti-Ig plus HRP-DAB Novolink polymer detection system (Leica). Tissue sections were analyzed using a laser-scanning microscope (Hamamatsu; nano Zoomer S60).

### Reverse transcription and quantitative polymerase chain reaction (RT-qPCR)

After treatment with different stimulants, mRNA was extracted from cells using TRIZOL® Reagent (Invitrogen Life Technologies, Carlsbad, CA, USA). cDNA was obtained from 2,5 µg of total mRNA, using Reverse Transcription kit (All-in-one 5X RT Master Mix, ABM). Quantitative real-time PCR analysis of the targeted genes was done using the specific primers (listed in S1 Table), 100 ng of cDNA and following manufacturer’s instructions, with a melting temperature at 60°C, previously determined by gradient PCR (BlasTaq™ 2X qPCR MasterMix, ABM). Relative mRNA levels were calculated by normalizing to *GAPDH* mRNA using 2^-ΔΔCt^ method.

### Single cells analysis

Using data of scRNAseq based on IgAN public dataset [37] (4 IgAN and 3 control patients) by bioinformatic analysis, we identified and compared subclusters of cells from cIgAN patients and control biopsies to identify complete transcriptomic signature. The controls were patients with non-autoimmune kidney diseases, including thin basement membrane disease and CAKUT. Using CellPhoneDB, we identified the different cell types and conduct a cell-cell interaction analysis based on the identification of ligand-receptor co-expression [38]. We particularly analyzed the expression of mTOR and TfR1 in different types of cells.

### Statistical analysis

Normality was assessed with the Shapiro-Wilk test, while differences between groups were evaluated using the Mann-Whitney and Fisher exact tests, as appropriate. For analysis involving multiple comparisons, either analysis of variance or the Kruskal-Wallis test was applied. Outliers were detected and removed based on the Dixon test. Correlations were measured using the Spearman rank correlation coefficient. All tests applied were two-sided, with a P-value threshold of less than 0.05 for statistical significance. *: P ≤ 0.05, **: P ≤ 0.01, ***: P ≤ 0.001****: P ≤ 0. 0001.Statistical computations were carried out using GraphPad Prism, version 10.

## Results

### TfR1 and mTOR are overexpressed in mesangial cells from IgAN patients

**Fig 1:**
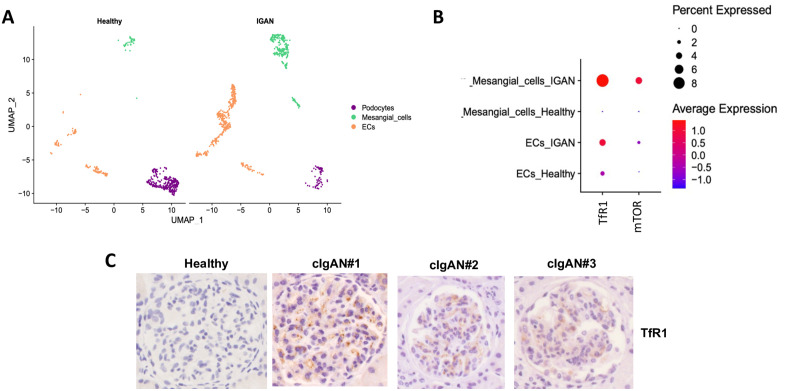
TfR1 and mTOR are overexpressed in human mesangial cells from IgAN patients during single cell analysis and in kidney biopsies of childhood IgAN (cIgAN). **(A)** Distribution of cell types in the glomeruli of healthy and IgAN patients. Endocapillary cells (Ecs). **(B)** Dot plot showing gene expression in luminal mesangial cells of TfR1 receptor and mTOR after scRNAseq analysis. **(C)** Mesangial TfR1 is overexpressed in kidneys from cIgAN patients compared to healthy control. Data were obtained by immunohistochemistry in childhood biopsies.

Single cell analysis of kidneys from the IgAN public dataset [37] (4 IgAN patients and 3 control patients) revealed an increase of mesangial cell numbers in IgAN patients (Fig 1A). This cell subtype showed major increases in *TfR1* and *mTOR* expression as observed in scRNAseq analysis (Fig 1B). Moreover, we detected TfR1 overexpression in the glomerular area by immunostaining in three cIgAN kidney biopsies. These biopsies displayed mesangial expansion, endocapillary hypercellularity, and cellular crescent, whereas the control kidney biopsy was negative for TfR1 staining in the glomerular area (Fig 1C).

### rsCD89 induces mTOR, Akt and S6K1 phosphorylation in human mesangial cells

**Fig 2:**
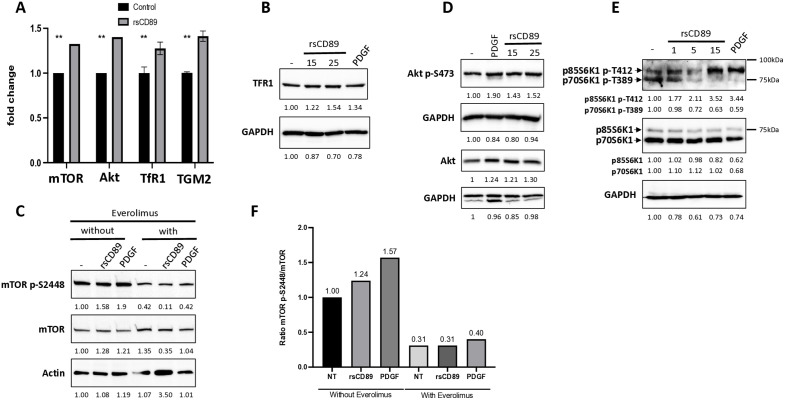
Recombinant soluble CD89 (rsCD89) modulates mTOR and Akt pathways through TfR1 expression in human mesangial cells (HMCs). **(A)** Quiescent HMCs were stimulated with rsCD89 (15 μg/ml), with β-synuclein used as a negative control. rsCD89 induced the expression of *mTOR*, *AKT*, *TfR1*, and *Transglutaminase 2* (*TGM2*) as measured by RT-qPCR. **(B)** TfR1 protein expression was upregulated by rsCD89. **(C)** rsCD89 stimulation induces phosphorylation of mTOR at S2448 (mTOR p-S2448/mTOR). **(D)** rsCD89 stimulation induced phosphorylation of downstream Akt at S473 **(E)** rsCD89 stimulation induced phosphorylation of the p85 isoform of S6K1 at T412. **(F)** Everolimus treatment of HMCs for 1 hour decreased mTOR phosphorylation at S2448, even in the presence of rsCD89 or PDGF.

To examine whether sCD89-TfR1 interaction induces mTOR activation in mesangial cells, we performed *in vitro* experiments stimulating HMCs with rsCD89 in absence of IgA at different time points (20 min, 35 min and 1 hour). rsCD89 (15 μg/ml) directly increased mRNA expression of *TfR1*, *mTOR*, *AKT* and *Transglutaminase 2* (*TGM2*) (Fig 2A). At protein level, rsCD89 induced an increase in TfR1 expression (Fig 2B) followed by phosphorylation changes of mTOR (mTOR p-S2448/mTOR) (Figs 2C and F left panels), but also of Akt (p-S473 Akt) (Fig 2D). Notably, rsCD89 differentially regulated S6K1 isoforms: phosphorylation of the p70 isoform S6K1 (p70S6K1 p-T389) decreased, whereas that of the p85 isoform (p85S6K1 p-T412, detected by the same antibody) increased (Fig 2E). Everolimus treatment of HMCs for 1 hour resulted in marked decrease mTOR phosphorylation at S2448 (mTOR p-S2448/mTOR), even in the presence of rsCD89 or PDGF (Figs 2C and 2F right panel).

### Everolimus prevents IgAN development in the α1KI mice injected with rsCD89

**Fig 3:**
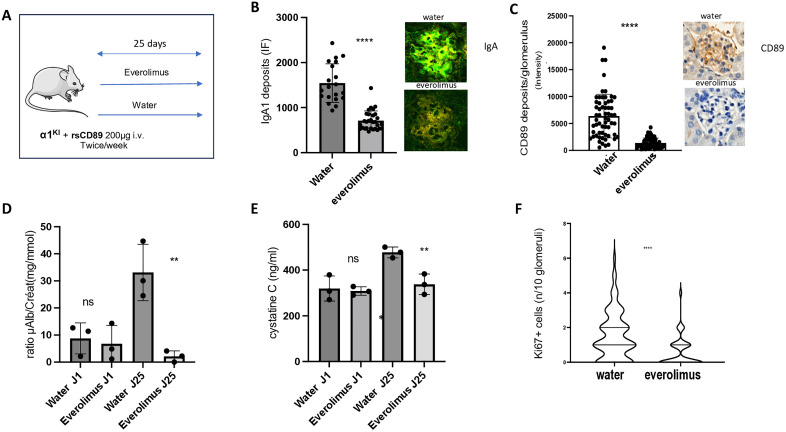
Everolimus prevents IgAN development in the α1^KI^ mice injected by rsCD89. (A). Experimental setup using α1^KI^ mice injected with rsCD89 twice a week. These mice were used to test everolimus as an effective treatment with mTOR inhibitors (2 mg/kg/day;) compared to vehicle for 25 days. Quantification of immunostainings were performed in 10 glomeruli per mouse studied as follows: (B). Glomerular IgA1 deposits in immunofluorescence (IF). (C). Glomerular sCD89 deposits in immunohistochemistry. (D). Proteinuria (ratio microalbuminuria (μAlb)/creatinuria mg/mmol). (E). Serum cystatin C levels (ng/ml). (F). Ki67 immunostaining in kidney mice. Representative immunostaining data from water- and everolimus-treated mice are shown on the right side of each panel for IgA1 (green) (B) and sCD89 (brown) (D) (mice # 1 and #4, respectively).

To explore whether mTOR inhibitors could block sCD89-mediated effects in mesangial cells in vivo, we first choose a shorter therapy in the α1^KI^ mice injected with rsCD89 that is able to induce the IgAN disease, as described previously [21,31]. Six α1^KI^ mice received 200 μg rsCD89 twice a week over four weeks, with three of them treated with everolimus (2 mg/kg) and three receiving water as a control (Fig 3A). Prior to treatment, IgA1 levels, proteinuria (microalbuminuria (μAlb)) and cystatin C levels were comparable between the groups. After 25 days (D25) everolimus decreased glomerular IgA1 deposits and mesangial sCD89 deposits (Figs 3B and C, and S1 Fig), reduced proteinuria levels (Fig 3D), while renal function remained stable as evaluated by cystatin C levels whereas renal function deteriorated in the placebo group (Fig 3E). Everolimus decreased also Ki67 staining in kidney (Fig 3F). The levels of Gd-IgA1 were not affected by the treatment (S2A Fig).

### Everolimus reverts IgAN phenotype and halts disease progression in the α1KICD89Tg mice

**Fig 4:**
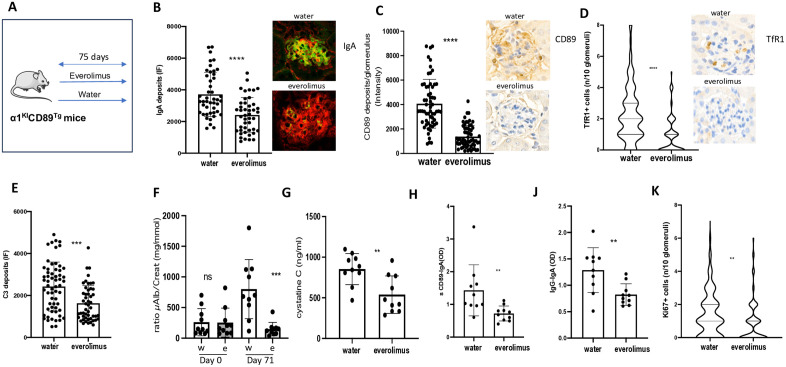
In vivo inhibition of mTOR pathway reverses IgAN progression in the α1^KI^CD89^Tg^ mice. (A) 8-week-old α1^KI^CD89^Tg^ mice expressing human IgA1 and CD89 were used for testing everolimus as effective treatment with mTOR inhibitors (2 mg/kg/day; 10 mice) as compared to vehicle (10 mice) for 75 days. Quantification of immunostainings were performed in 10 glomeruli per mouse at the end of the treatment (B) IgA1 deposits, (C) sCD89 deposits, (D) quantification of TfR1 expression and (E) C3 deposits. Representative immunostaining data from water- and everolimus-treated mice are shown on the right side of each panel for IgA1(green) (B), sCD89 (brown) (C) and TfR1 (brown) (D) (mice # 1 and #11, respectively). (F) Proteinuria (ratio microalbuminuria (μAlb)/creatinuria mg/mmol) before and after treatment. (G) Serum cystatin C levels at day 75. (H) Serum sCD89-IgA complexes at day 75. (J) Serum IgG-IgA complexes at day 75. (K) Quantification Ki67 staining. Data are shown as mean ± standard error of mean. Statistical significance was determined using a nonparametric Mann-Whitney test or Spearman rank correlation coefficients. (*) p < 0.05, P < 0.05 and ***P < 0.001 compared with the control group. OD, optical density ns (non-significant).

To validate the mTOR inhibitor effect in mice spontaneously developing IgAN-like disease, we took advantage of the α1^KI^CD89^Tg^ mouse model that progress with time by developing mesangial expansion and IgA1 and sCD89 deposits associated with proteinuria and hematuria [31]. To treat initial IgAN model (that might mimic cIgAN disease), ten 8 w-old α1^KI^CD89Tg [31] mice were treated daily with everolimus by intragastric gavage for 75 days, while another ten mice received water as a vehicle (Fig 4A). At the endpoint all water-treated animals showed progressive disease as previously described [31]. In contrast, everolimus markedly decreased IgA1 deposits in glomeruli (Fig 4B and S3 Fig) and sCD89 (Fig 4C and S4 Fig) as well as mesangial TfR1 expression (Fig 4D and S5 Fig). Moreover, everolimus-treated group exhibited a significant reduction in C3 deposits, proteinuria (microalbuminuria(μAlb)) and serum cystatine C (Figs 4E-G), which were associated with a decrease expression of serum levels of sCD89-IgA1 and IgG-IgA1 complexes (Figs 4H-J). Gd-IgA1 levels did not reach statistical significance (S2B Fig). Everolimus induced decreased kidney hypercellularity as quantified by Ki67 immunostaining (Fig 4K).

## Discussion

The main finding of our work is that mTOR inhibition ameliorates IgA nephropathy in humanized mice by correcting CD89 and CD71 dysfunctions. This effect is associated with reduced IgA and C3 deposits, decreased proteinuria (microalbuminuria, μAlb), and lower serum cystatin C levels. Our study highlights the pivotal role of CD89 and CD71 in IgAN pathogenesis through the mTOR pathway.

In the present study, we first utilized publicly available single cell analysis data [37] to show that *TfR1* (*CD71*) mRNA is overexpressed in mesangial cells from four patients with IgAN confirming our previous observations [26,28]. This overexpression was associated with increased mTOR expression. Immunohistochemical analysis of kidney biopsies from cIgAN patients revealed CD71 overexpression in mesangial cells confirming data previously observed in adult IgAN and in HSP at the protein level [26,28]. Since sCD89 was previously shown to interact with CD71 mesangial receptor [21,31], these observations led us to evaluate the effect of sCD89 on cultured mesangial cells. Our results reveal that sCD89 directly induces upregulation of CD71 and activation of mTOR pathway. Indeed, phosphorylated adaptors/kinases such as mTOR, Akt and the less well-characterized p85 isoform of S6K1 [39] were observed following sCD89 triggering of CD71.

Recent biopharma efforts provided new therapeutic approaches leading to health care progress for patients with IgAN [1,2,40]. They included mostly B cell and complement modulators but also a spleen tyrosine kinase (SYK) inhibitor [41]. However, despite of promising results in phase 2 and 3 RCT trials, only partial responses were achieved without complete remission. Moreover, no clinical trials exist for these therapies in pediatric populations, limiting treatment options and leaving corticosteroids and renin angiotensin blockers treatment as the primary approach. Direct targeting of IgA receptor signaling pathways in mesangial cells remains poorly explored as a strategy to prevent or inactivate the progression of the disease.

IgA receptors are essential effectors involved in IgA-immune responses. Among several IgA receptors, two of them have been shown to be associated with inflammatory diseases [42], notably in IgAN [43]. CD89 is a transmembrane IgA Fc receptor expressed on human myeloid cells, while CD71 functions as an alternative receptor for IgA1 [42]. CD71 is highly expressed on mesangial cells and enterocytes primarily under pathological conditions associated with IgAN, Henoch-Schönlein purpura (HSP), and celiac disease [26,27,42]. While CD71 binds IgA1, depending on its expression level and on IgA1 glycosylation [29], CD89 can bind both IgA1 and IgA2. However, CD89 soluble form (sCD89) is not commonly found in the circulation of healthy individuals [31]. Under healthy conditions, myeloid CD89 regulates inflammatory responses notably through interaction with monomeric IgA (mIgA), the second most abundant Ig isotype in serum, that induces an anti-inflammatory effect via tyrosine phosphatase recruitment [44,45]. In contrast CD89 binding to polymeric IgA (pIgA) or IgA-containing immune complex aggregates leads to the recruitment of the SYK kinase, triggering pro-inflammatory responses [43,44].

It has previously been demonstrated that cIgAN patients exhibit two types of IgA1-containing CICs. While large CICs stimulate proliferation of cultured HMCs whereas small complexes inhibit cellular proliferation [46]. However, the composition of stimulatory CICs other than Gd-IgA1 has not been investigated, notably the role of sCD89. In cIgAN patients, as in adult patients, decreased levels of cell surface CD89 in blood monocytes and neutrophils have been observed probably due to a shedding mechanism involving proteases [16,21] which is associated with the presence of sCD89 in the systemic blood of IgAN patients [14]. Interestingly, it has been shown that decrease in sCD89 levels in the blood is associated with severe cases and with IgAN progression towards renal failure in adults [47]. This was confirmed in patients with recurrent IgA deposits after renal transplantation [19]. In cIgAN, sCD89 binds pathological Gd-IgA1 with higher affinity than physiological mIgA1, promoting pro-inflammatory responses and mesangial IgA1 deposition [48]. We recently demonstrated the pivotal role of sCD89 by inducing mesangial cell proliferation in cIgAN. The injection of recombinant sCD89 into transgenic mice expressing human IgA1 induced glomerular inflammation and mesangial cell proliferation [21]. Moreover, plasma levels of sCD89 and sCD89-IgA1 in cIgAN patients positively correlated with proteinuria, estimated glomerular filtration rate (eGFR), and with glomerular inflammation M1, E1, C1, and S1[21]. Also, sCD89 and sCD89-IgA1 levels were more sensitive and specific than proteinuria in predicting glomerular inflammation, endocapillary hypercellularity, and cellular crescents in cIgAN [21].

Similar to CD89, TfR1 (CD71) can also bind polymeric IgA1 and IgA1-containing immune complexes that are able to induce activation of mesangial cells through ERK-PI3K-mTOR signaling pathway leading to receptor overexpression and cell proliferation [21,30–32]. However, CD71 preferentially binds Gd-IgA1 rather than normally glycosylated IgA1 that may explain why normally glycosylated monomeric IgA1 cannot induce mesangial receptor alterations in healthy individuals [29].

mTOR inhibitors are commonly used in clinics [49]. The mTOR inhibitor sirolimus was previously studied in a non-controlled trial as a potential treatment in 23 adult patients with IgAN showing stabilization of renal function, reducing glomerular proliferative lesions. Nevertheless, the interpretation of these results was troubled by its association with another drug, the enalapril [50]. Moreover, since 2011 no trial has been performed probably due to the lack of a clear rationale for a mechanism of action in IgAN. We evaluated the effect of everolimus using two models of IgAN, the young 1^KI^ mice injected with rsCD89 and the spontaneous IgAN model the 1^KI^CD89^Tg^ mice. In both models, the drug was able either to prevent or to revert established IgAN disease with marked decrease in human IgA1 and sCD89 deposits, mouse C3 deposits, mouse TfR1 (CD71) expression, proteinuria, cystatin C, and the Ki67 proliferation marker. The observed decrease in TfR1 expression and CD89 deposition by everolimus is particularly intriguing. A potential negative feedback mechanism may be driven by the reduction of IgA1 and sCD89 deposits following mTOR inhibition, suggesting a regulatory loop involving IgA1, CD89, and TfR1. A plausible explanation could involve a transcriptional mechanism, as mTOR signaling activates transcription factors such as HIF and MYC, both known to enhance TfR1 transcription [51]. Alternatively, a post-transcriptional mechanism could contribute, whereby mTOR activation inhibits its downstream target, tristetraprolin, stabilizing TfR1 mRNA and prolonging its expression [52]. Inhibition of mTOR by everolimus could therefore downregulate TfR1 expression, disrupting the sCD89–TfR1 interaction and ultimately reducing sCD89 deposition.

This agrees with previous data showing that the rapamycin effect, another mTOR inhibitor, is able to reduce proteinuria and kidney deposition of rat IgA in an IgAN model developed in rats [53]. However, mTOR inhibition does not appear to significantly impact systemic or mucosal IgA1 production, as circulating levels of Gd-IgA1 remained largely unchanged, with only a slight decrease observed in the everolimus-treated group compared to controls. This suggests that mTOR inhibition primarily affects IgA1 deposition within the kidney rather than its upstream production. It is also likely that everolimus does not influence the gut microbiota, particularly mucin-degrading bacteria, which have been implicated in Gd-IgA1 production in this mice model. [54] These findings highlight the importance of further investigating mesangial IgA receptor signaling in IgAN and its modulation by mTOR inhibition. Moreover, these findings underscore the need for future clinical trials to validate the efficacy and safety of mTOR inhibitors in human IgAN, particularly in pediatric patients, where corticosteroids remain the only approved immunosuppressive treatment.

## Conclusion

IgA1 and/or sCD89 activate mesangial cells via CD71-mTOR pathway. This study provides a pre-clinical assay for mTOR inhibitor, the everolimus, as attractive therapeutic option in IgAN. It supports novel trials in IgAN, notably in cIgAN where effective treatment targeting mesangial IgA receptors remains poorly provided.

## Supporting information

S1 Fig Everolimus reduce IgA1 (green) and sCD89 (brown) deposits in the a1KI model after injection of rsCD89. α1 ^KI^ mice injected with rsCD89 twice a week. These mice were used to test everolimus as an effective treatment with mTOR inhibitors (2 mg/kg/day;) compared to vehicle for 25 days. Glomeruli were stained with anti-human IgA1 antibodies (green) using immunofluorescence and with monoclonal antibody anti-CD89 in immunohistochemistry (brown). # indicates mice numbers.
(TIF)

S2 Fig No effect observed on serum Gd-IgA1 levels by everolimus in both IgAN models. (TIF)

S3 Fig Everolimus decreased IgA1 deposits in glomeruli of α1^KI^CD89^Tg^ mice. 8-week-old α1^KI^CD89^Tg^ mice expressing human IgA1 and CD89 were used for testing everolimus as effective treatment with mTOR inhibitors (2 mg/kg/day; 10 mice) as compared to vehicle (10 mice). Glomeruli were stained with anti-human IgA1 antibodies (green) using immunofluorescence. Representative images of 20 glomeruli (#2–#10 from the vehicle-treated group and #12–#20 from the everolimus-treated group) demonstrate a significant reduction in IgA1 deposits in the glomeruli of mice treated with everolimus compared to those treated with the vehicle. # indicates mice numbers.
(TIF)

S4 Fig Everolimus decreased CD89 deposits in α1^KI^CD89^Tg^ mice. 8-week old α1^KI^CD89^Tg^ mice expressing human IgA1 and CD89 were used for testing everolimus as effective treatment with mTOR inhibitors (2 mg/kg/day; 10 mice) as compared to vehicle (10 mice). Glomeruli were stained with monoclonal antibody anti-CD89 in immunohistochemistry (brown). Representative images of 20 glomeruli (#2–#10 from the vehicle-treated group and #12–#20 from the everolimus-treated group) demonstrate a significant reduction in CD89 deposits in the glomeruli of mice treated with everolimus compared to those treated with the vehicle. # indicates mice numbers.
(TIF)

S5 Fig Everolimus decreased mesangial TfR1 expression in α1^KI^CD89^Tg^ mice. 8-week old α1^KI^CD89^Tg^ mice expressing human IgA1 and CD89 were used for testing everolimus as effective treatment with mTOR inhibitors (2 mg/kg/day; 10 mice) as compared to vehicle (10 mice). Glomeruli were stained with monoclonal antibody anti-TfR1 in immunohistochemistry (brown). Representative images of 20 glomeruli (#2–#10 from the vehicle-treated group and #12–#20 from the everolimus-treated group) demonstrate a significant reduction in mesangial TfR1 expression in the glomeruli of mice treated with everolimus compared to those treated with the vehicle. # indicates mice numbers.
(TIF)

S1 Table Oligonucleotides used in this work. (DOCX)

S1 File Raw image. (PDF)
