# Correction: Blockade of mTOR ameliorates IgA nephropathy by correcting CD89 and CD71 dysfunctions in humanized mice

**DOI:** 10.1371/journal.pone.0345118

**Published:** 2026-03-17

**Authors:** Alexandra Cambier, Jennifer Da Silva, Julie Bex, Fanny Canesi, Lison Lachize Neanne, Aurélie Sannier, Hélène Mathieu, Erwan Boedec, Amandine Badie, Renato C. Monteiro

The fifth author’s initials appear incorrectly in the citation. The correct citation is: Cambier A, Da Silva J, Bex J, Canesi F, Lachize Neanne L, Sannier A, et al. (2025) Blockade of mTOR ameliorates IgA nephropathy by correcting CD89 and CD71 dysfunctions in humanized mice. PLoS One 20(10): e0318581. https://doi.org/10.1371/journal.pone.0318581.

A second affiliation for the fifth author is not indicated. Lison Lachize Neanne is also affiliated with: Axe Maladies immunitaires et cancer, Centre de Recherche Azrieli, Université de Montréal, CHU Sainte-Justine, Montréal, Canada.

## References

[1] Cambier A, Da Silva J, Bex J, Canesi F, Neanne LL, Sannier A, et al. Blockade of mTOR ameliorates IgA nephropathy by correcting CD89 and CD71 dysfunctions in humanized mice. PLoS One. 2025;20(10):e0318581. doi: 10.1371/journal.pone.0318581 41056297 PMC12503266

