# Supplementary figures and images for: Blockade of mTOR ameliorates IgA nephropathy by correcting CD89 and CD71 dysfunctions in humanized mice
Source: PLoS One. 2025 Oct 7;20(10):e0318581. doi: 10.1371/journal.pone.0318581 (PMC12503266; doi:10.1371/journal.pone.0318581)

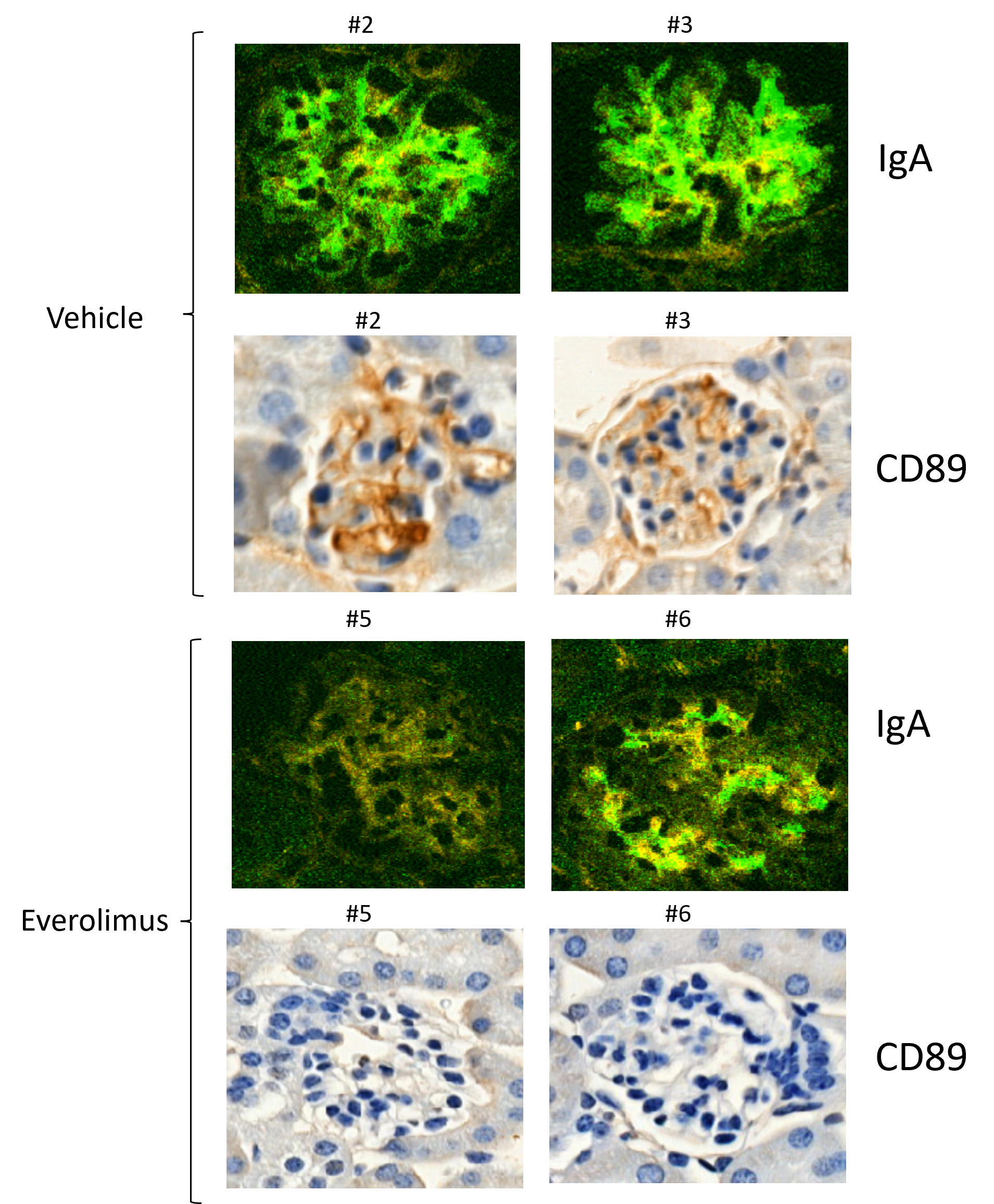

Supplement: S1 Fig — These mice were used to test everolimus as an effective treatment with mTOR inhibitors (2 mg/kg/day;) compared to vehicle for 25 days. Glomeruli were stained with anti-human IgA1 antibodies (green) using immunofluorescence and with monoclonal antibody anti-CD89 in immunohistochemistry (brown). # indicates mice numbers. (TIF) [file pone.0318581.s001.tif]

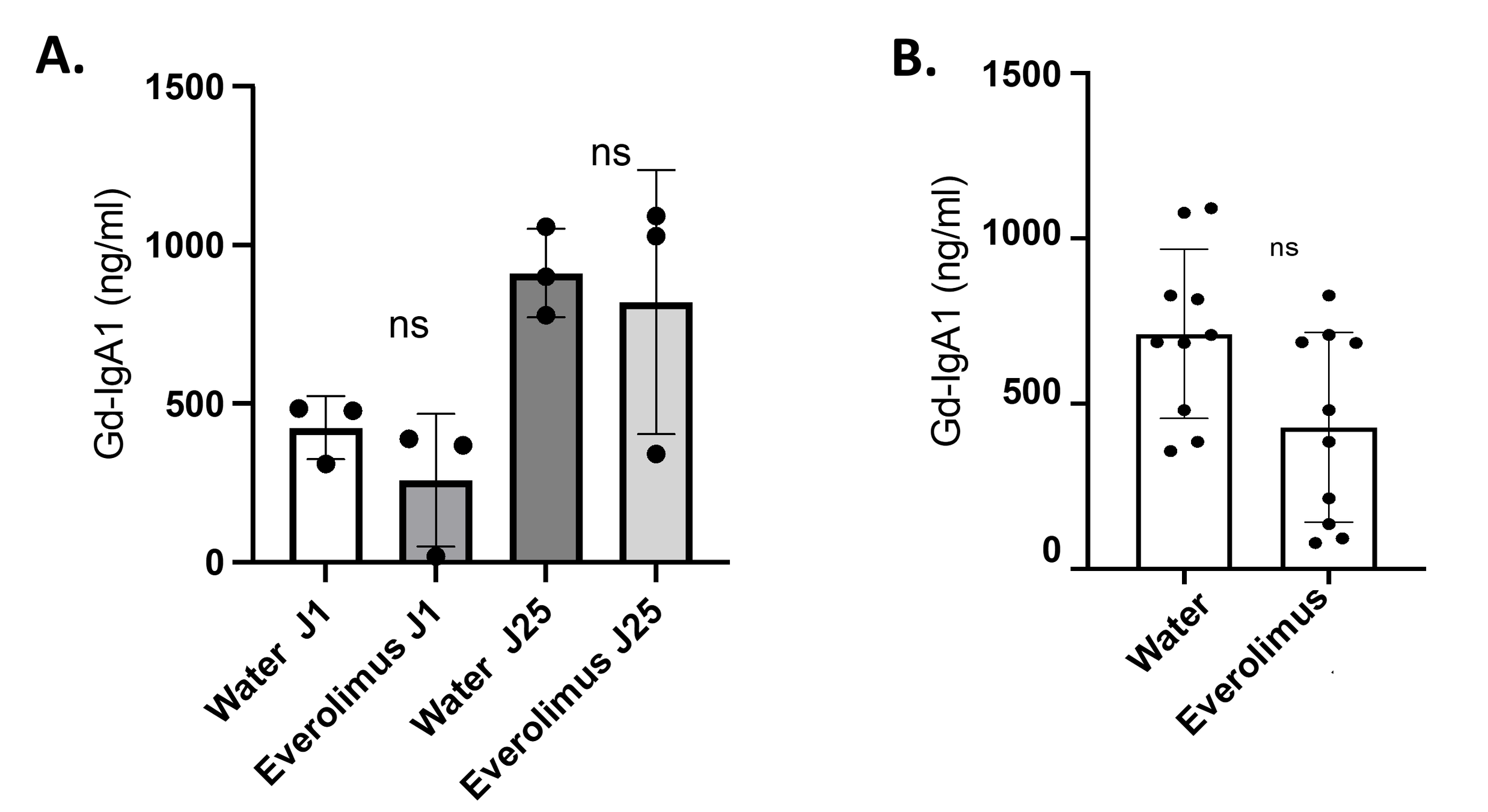

Supplement: S2 Fig — (TIF) [file pone.0318581.s002.tif]

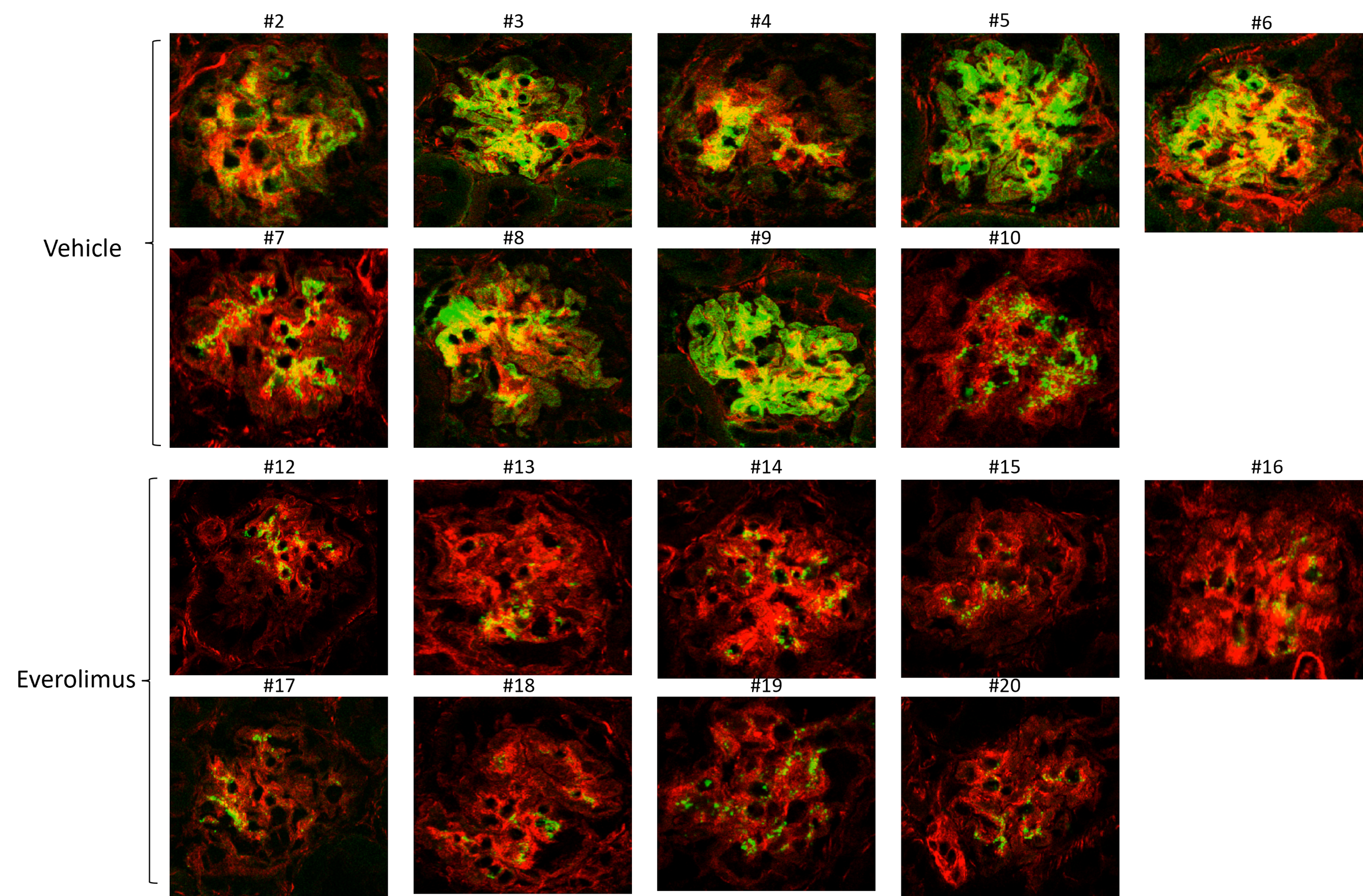

Supplement: S3 Fig — 8-week-old α1KICD89Tg mice expressing human IgA1 and CD89 were used for testing everolimus as effective treatment with mTOR inhibitors (2 mg/kg/day; 10 mice) as compared to vehicle (10 mice). Glomeruli were stained with anti-human IgA1 antibodies (green) using immunofluorescence. Representative images of 20 glomeruli (#2–#10 from the vehicle-treated group and #12–#20 from the everolimus-treated group) demonstrate a significant reduction in IgA1 deposits in the glomeruli of mice treated with everolimus compared to those treated with the vehicle. # indicates mice numbers. (TIF) [file pone.0318581.s003.tif]

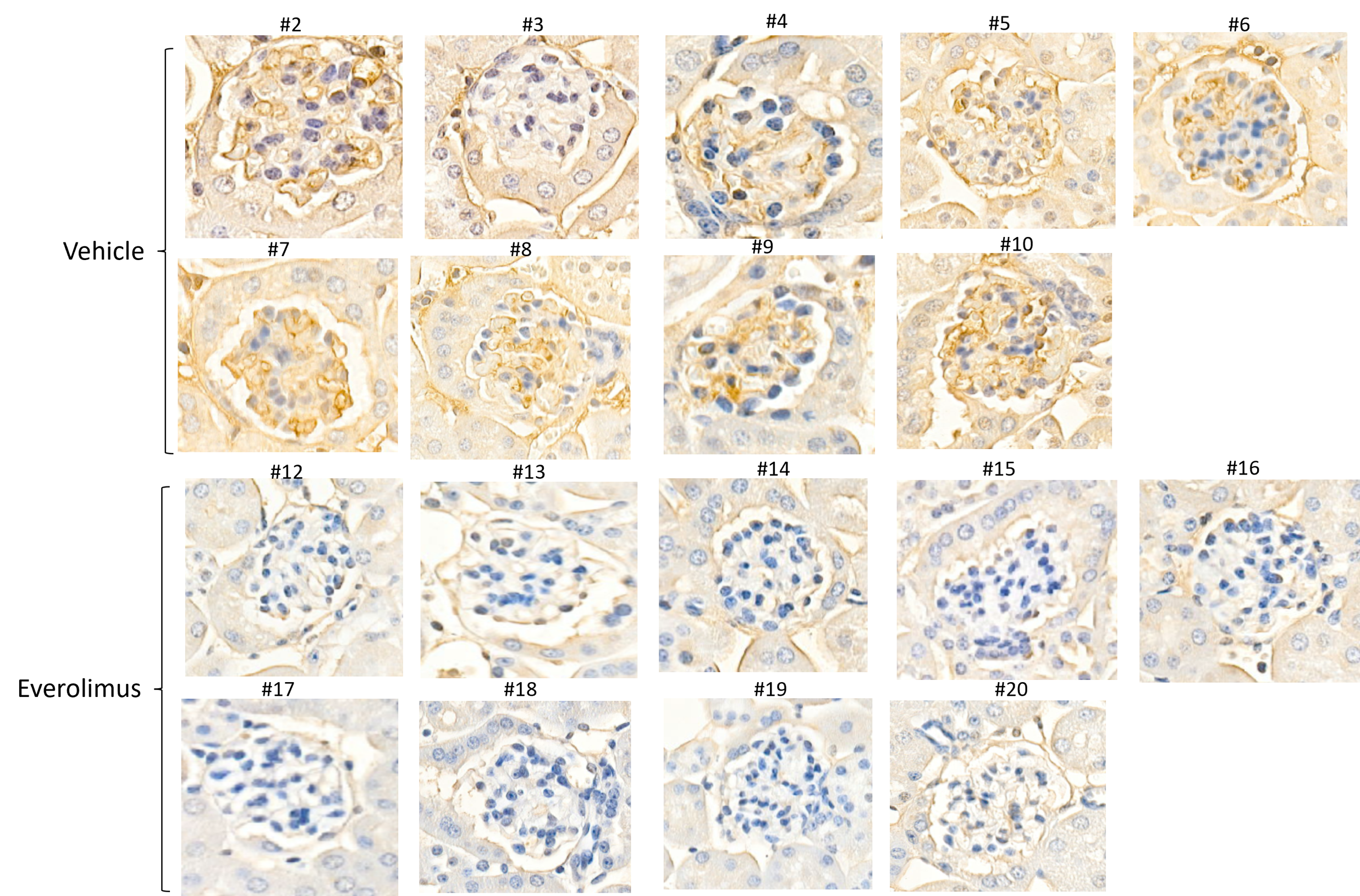

Supplement: S4 Fig — 8-week old α1KICD89Tg mice expressing human IgA1 and CD89 were used for testing everolimus as effective treatment with mTOR inhibitors (2 mg/kg/day; 10 mice) as compared to vehicle (10 mice). Glomeruli were stained with monoclonal antibody anti-CD89 in immunohistochemistry (brown). Representative images of 20 glomeruli (#2–#10 from the vehicle-treated group and #12–#20 from the everolimus-treated group) demonstrate a significant reduction in CD89 deposits in the glomeruli of mice treated with everolimus compared to those treated with the vehicle. # indicates mice numbers. (TIF) [file pone.0318581.s004.tif]

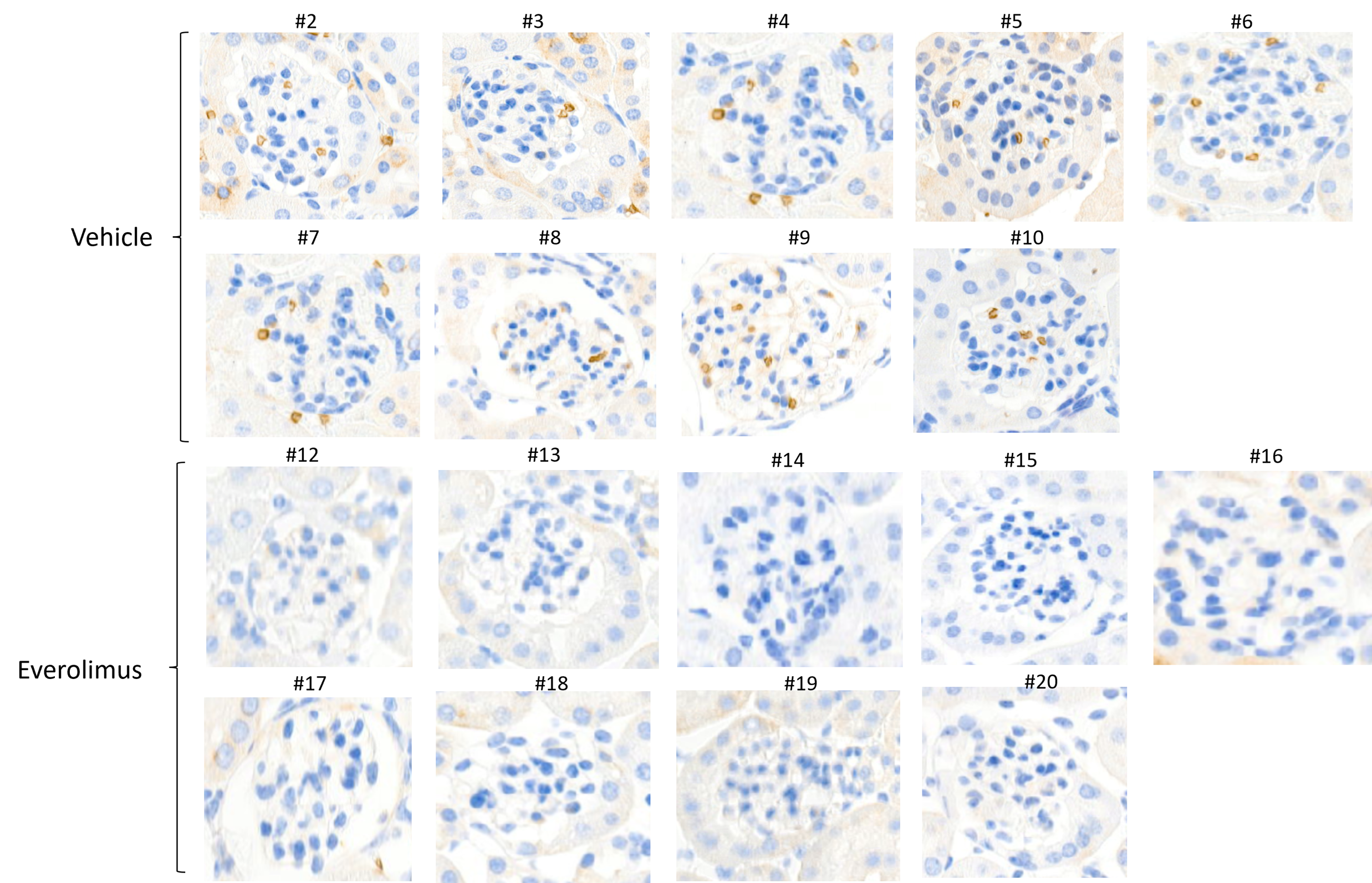

Supplement: S5 Fig — 8-week old α1KICD89Tg mice expressing human IgA1 and CD89 were used for testing everolimus as effective treatment with mTOR inhibitors (2 mg/kg/day; 10 mice) as compared to vehicle (10 mice). Glomeruli were stained with monoclonal antibody anti-TfR1 in immunohistochemistry (brown). Representative images of 20 glomeruli (#2–#10 from the vehicle-treated group and #12–#20 from the everolimus-treated group) demonstrate a significant reduction in mesangial TfR1 expression in the glomeruli of mice treated with everolimus compared to those treated with the vehicle. # indicates mice numbers. (TIF) [file pone.0318581.s005.tif]

Figure 2B

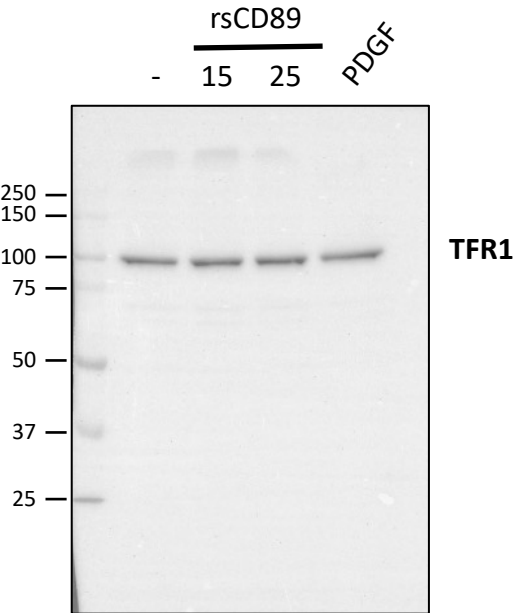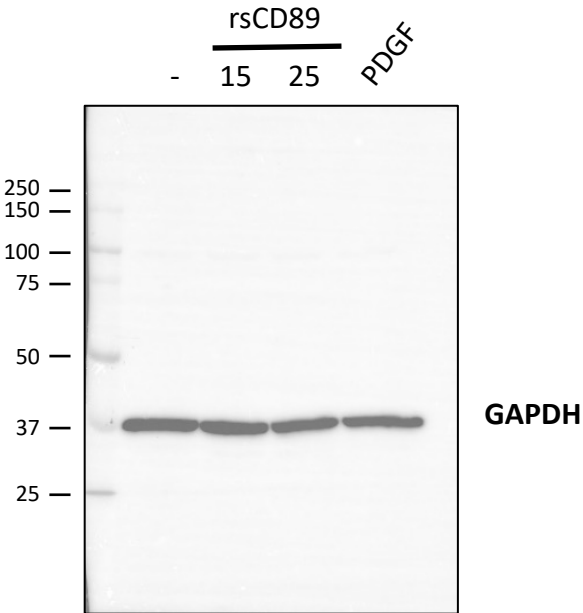

Figure 2C

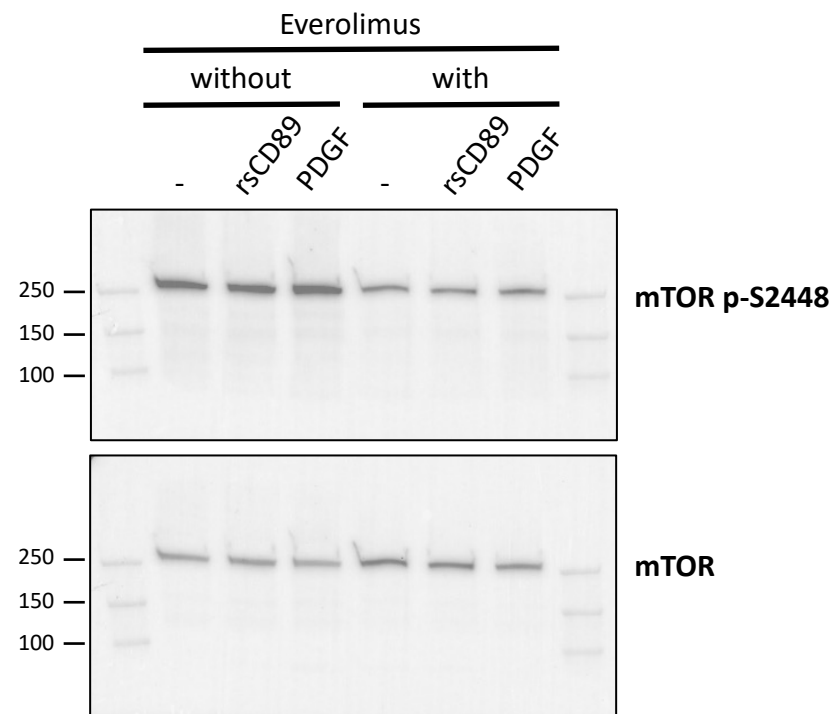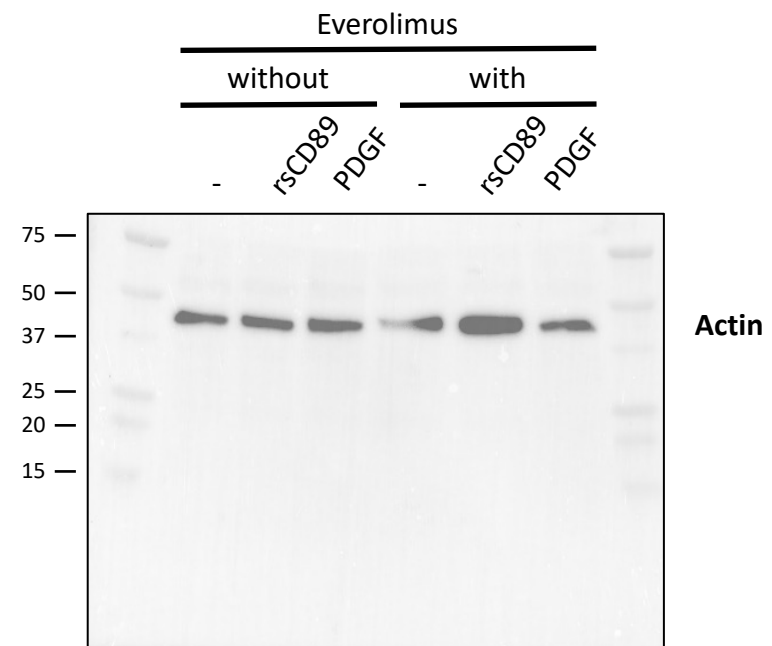

Figure 2D

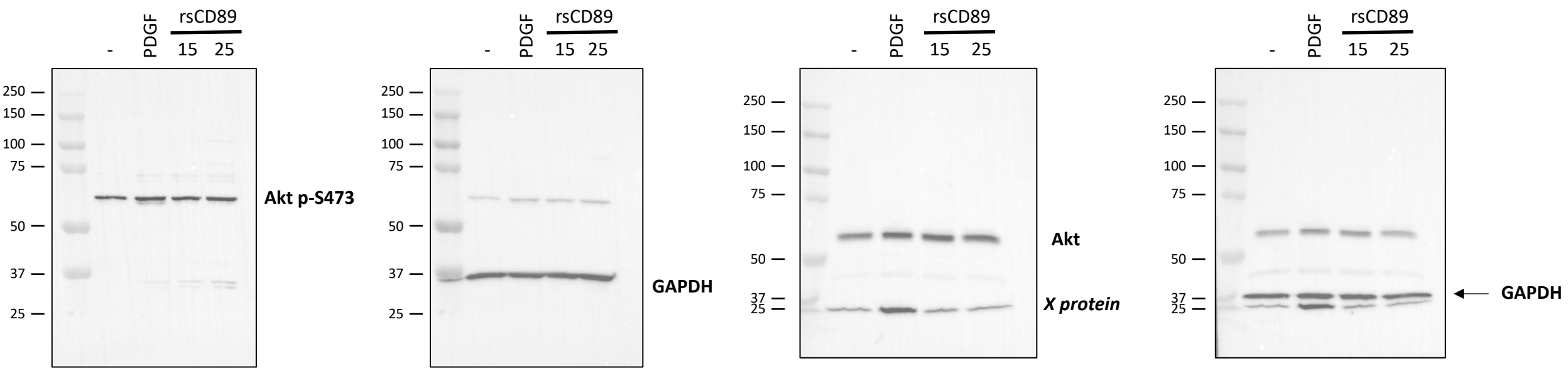

Figure 2E

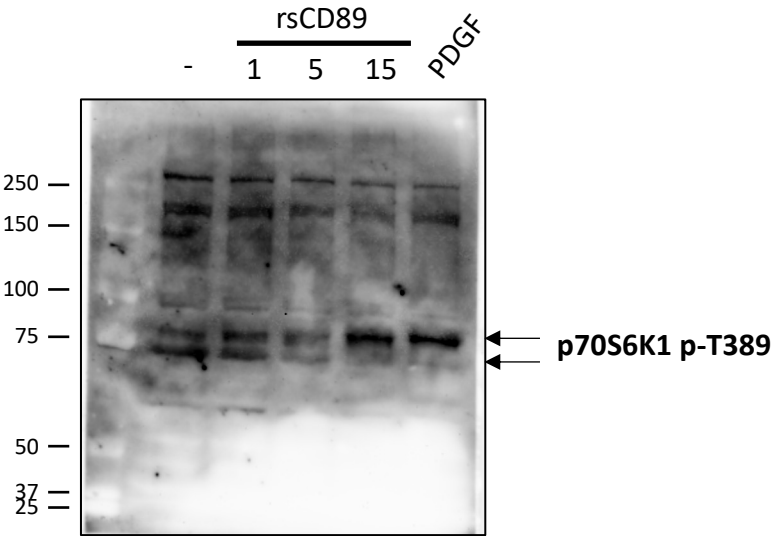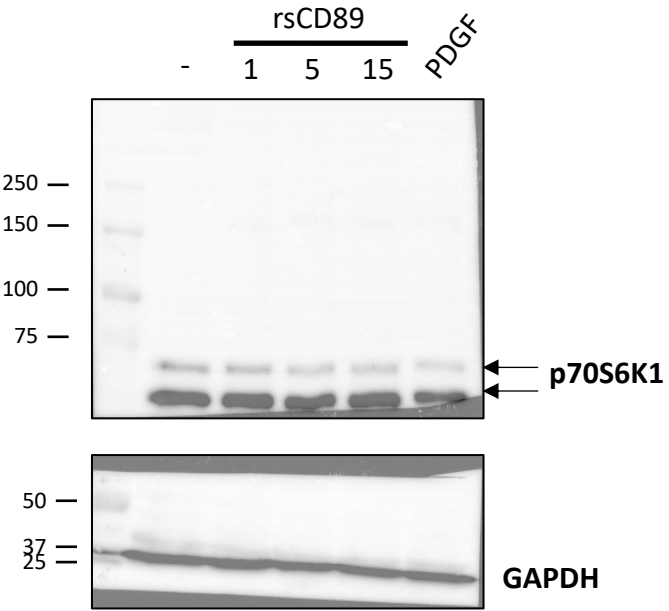

Supplement: S1 File — (PDF) [file pone.0318581.s007.pdf]
