# Supplementary material for: Blockade of mTOR ameliorates IgA nephropathy by correcting CD89 and CD71 dysfunctions in humanized mice
Source: PLoS One. 2025 Oct 7;20(10):e0318581. doi: 10.1371/journal.pone.0318581 (PMC12503266; doi:10.1371/journal.pone.0318581)
Supplement: S1 Table — (DOCX) [file pone.0318581.s006.docx]

S1 Table: Oligonucleotides used in this work

| **Name** | **Sequence** |
| --- | --- |
| Fw GAPDH | TGCACCACCAACTGCTTAGC |
| Rv GAPDH | GGCATGGACTGTGGTCATGAG |
| Fw TgM2 | ATGTCAACCCCAAGTTCCTG |
| Rv TgM2 | CCGTCCCCGTAGTTGTTGT |
| Fw TfR1 | TCTCGGTCATCAGGATTGCC |
| Rv TfR1 | GACAGTCTCCTTCCATATTCCC |
| Fw AKT | TTCTGCAGCTATGCGCAATGTG |
| Rv AKT | TGGCCAGCATACCATAGTGAGGTT |
| Fw mTOR | GCTTGATTTGGTTCCCAGGACAGT |
| Rv mTOR | GTGCTGAGTTTGCTGTACCCATGT |
